# IgA Antibodies and IgA Deficiency in SARS-CoV-2 Infection

**DOI:** 10.3389/fcimb.2021.655896

**Published:** 2021-04-06

**Authors:** Isabella Quinti, Eva Piano Mortari, Ane Fernandez Salinas, Cinzia Milito, Rita Carsetti

**Affiliations:** ^1^ Department of Molecular Medicine, Sapienza University of Rome, Rome, Italy; ^2^ Department of Laboratory Medicine, Research Area Multimodal Medicine, Diagnostic Immunology and Research Unit, Bambino Gesù Children’s Hospital IRCCS, Rome, Italy

**Keywords:** IgA, secretory IgA, SARS-Cov-2, selective IgA deficiency, infectivity

## Abstract

A large repertoire of IgA is produced by B lymphocytes with T-independent and T-dependent mechanisms useful in defense against pathogenic microorganisms and to reduce immune activation. IgA is active against several pathogens, including rotavirus, poliovirus, influenza virus, and SARS-CoV-2. It protects the epithelial barriers from pathogens and modulates excessive immune responses in inflammatory diseases. An early SARS-CoV-2 specific humoral response is dominated by IgA antibodies responses greatly contributing to virus neutralization. The lack of anti-SARS-Cov-2 IgA and secretory IgA (sIgA) might represent a possible cause of COVID-19 severity, vaccine failure, and possible cause of prolonged viral shedding in patients with Primary Antibody Deficiencies, including patients with Selective IgA Deficiency. Differently from other primary antibody deficiency entities, Selective IgA Deficiency occurs in the vast majority of patients as an asymptomatic condition, and it is often an unrecognized, Studies are needed to clarify the open questions raised by possible consequences of a lack of an IgA response to SARS-CoV-2.

## Highlights

Lack of neutralizing anti-SARS-Cov-2 IgA and secretory IgA antibodies represents a possible cause of prolonged viral shedding in patients with Selective IgA Deficiency and in patients with Primary Antibody Deficiencies showing an increased length of SARS-CoV-2 positivity.

## Introduction

IgA is the predominant immunoglobulin in the respiratory tract, the major portal of entry for many microorganisms. There are two subclasses of IgA, IgA1 and IgA2. Monomeric IgA1 predominates in serum. Dimeric and polymeric IgA1 and IgA2 - linked by a J-chain - are present on the mucosal surface where they exert a major role in protection against toxins, viruses and bacteria by neutralization, or by preventing attachment to the mucosal epithelium. IgA1 predominates in the airways and IgA2 is predominant in the colon. IgA is produced by plasma cells of the submucosa and it is transported to the apical surface of intestinal epithelial cells by the efficient mechanism involving the polymeric immunoglobulin receptor (pIgR). On the basolateral surface of mucosal epithelial cells, the pIgR binds dimeric IgA through the J chain and transports the molecule to the apical cell membrane. Here, a serine-protease (Kaetzel, 2005) cleaves off the complex leaving the secretory component fragment (SC) of the pIgR attached to the immunoglobulin, thus generating sIgA. The importance of IgA comes from studies on patients with Primary Antibody Deficiencies (PAD), where the impaired IgA production, and IgA antibody-mediated responses, are associated to the susceptibility to respiratory and gastrointestinal infections and their recurrence (Hammarström et al., 2000; Tangye et al., 2020), to upper respiratory tract colonization (Pulvirenti et al., 2020a), and to the risk to develop chronic respiratory diseases (Quinti et al., 2011). The presence of sIgA with a diverse antigen-binding repertoire is essential for maintenance of mucosal protection of the upper and lower respiratory tracts, for mucosal immune responses by its interaction with mucosal epithelial cells, and by binding to antigens and cellular receptors (Cerutti et al., 2011). IgA shows a number of unique features among the immunoglobulin classes. IgA are monomeric, dimeric, and also polymeric, displaying different functions. Both antibody affinity and avidity are important in mechanisms of protection (Terauchi et al., 2018; Saito et al., 2019).

A large repertoire of IgA is produced by B lymphocytes with T-independent and T-dependent mechanisms useful in defense against pathogenic microorganisms and to reduce immune activation. IgA class switching is the process whereby B cells acquire the expression of IgA. The T-dependent process requires at least one week to develop, a too long time in mucosal infections. Natural antibodies, mostly of IgM isotype and generated independently of previous antigen encounters, have a broad reactivity and a variable affinity. They contain the infection during the 2 weeks necessary for production of high-affinity antibodies (Ochsenbein et al., 1999; Holodick et al., 2017).

Also IgA can be rapidly produced through the faster T-independent mechanisms involving B cell activating factor (BAFF) and its receptors. We have shown that most of the IgA in the gut is generated by a T-independent mechanism involving TLR9 and TACI from IgM memory B cells. Similar to natural serum IgM, “natural IgA” may function as immediate and early protection from infection (Carsetti et al., 2020a).

IgA plays a major role in protection against several viral pathogens including RSV and other influenza viruses. BAFF is increased in the lung and it is associated with the class-switched IgA antibody responses against RSV infection (McNamara et al., 2013), and BAFF neutralization results in reduced sIgA levels and AID expression during influenza virus infection (Wang et al., 2015). Local IgA responses, in cooperation with non-specific innate factors such as muco-ciliary clearance, have been shown to protect from influenza virus natural infection without inducing a potentially deleterious inflammatory response leading to tissue damage (Pilette et al., 2011). High levels of anti-influenza virus IgA in breast milk are associated with decreased infant episodes of respiratory illness (Schlaudecker et al., 2013).

The current challenge in vaccine design is to induce long-lasting systemic and mucosal protection against the vaccine strains, but also against drifted and shifted strains. Most antiviral vaccines are now administered intramuscularly or subcutaneously, and they might not always induce a mucosal immune response (van Riet et al., 2012; Bagga et al., 2015). It has been shown that IgA antibodies on mucosal surfaces play a more relevant role than IgG for protection from influenza A virus infection and for cross-protective immunity against multiple viral hemagglutinin subtypes (Okuya et al., 2020). More recently, it has been shown in mice that immunization with MERS-CoV S1 subunit and full-length Spike protein elicited high levels of S1-specific neutralizing IgA response. However, MERS-CoV neutralizing IgA antibodies in the broncho-alveolar lavage fluid were only induced by intranasal and sublingual administration but not by intramuscular administration (Kim et al., 2019).

## IgA Antibodies and SARS-CoV-2

Unless immunized, everyone is susceptible to SARS-CoV-2 infection. The variability of COVID-19 suggests that the individual immune response to SARS-CoV-2 may play a crucial role in determining the clinical course ranging from asymptomatic to mild upper respiratory tract illness, or moderate to severe disease with respiratory distress and multi-organ failure requiring intensive care and organ support (WHO, [[NoYear]]; Zhou et al., 2020). To pathogens for which there is no pre-existing immunity, our organism reacts by rapidly engaging the innate immune system with the intent of limiting the infection and giving time to adaptive immune response to generate the most specific and effective tools: high-affinity antibodies and memory B and T cells. Extensive analysis of the antibody response, showed that SARS-CoV-2 induces virus-specific antibodies, mediated by all immunoglobulin isotypes including IgM, IgG, and IgA (Long et al., 2020); all IgG subclasses were produced by individuals with COVID-19, with IgG1 being the most dominant (Goh et al., 2021). The kinetics of specific immunoglobulin production against spike-1 receptor-binding domain and nucleocapsid protein shows that the vast majority of patients produce detectable neutralizing IgG and IgA antibodies within 2-3 weeks from onset of symptoms. Then, neutralizing anti-RBD IgG and anti-NC titers increased and reached a plateau around the fourth week after symptom onset, while IgA decreased by day 28 (Sterlin et al., 2021). This might be observed also in asymptomatic SARS-CoV-2 infection as we have recently shown (Carsetti et al., 2020b).

IgA is active against several pathogens, including rotavirus, poliovirus, influenza virus, and SARS-CoV-2, it modulates excessive immune responses in inflammatory diseases and it is more effective in recruiting neutrophils for cell killing (Sterlin and Gorochov, 2021).

Peripheral expansion of IgA plasma blasts with mucosal-homing potential has been detected shortly after the onset of symptoms (Wang et al., 2020). The virus-specific antibody responses are mediated by all isotypes, but IgA contributes to virus neutralization to a greater extent compared with IgG and probably associated with protection against reinfection (Gaebler et al., 2021). During the first six months after infection, anti-SARS-CoV-2 memory B cell response evolves with accumulation of Ig somatic mutations. Sharing of VH sequences has been demonstrated between IgA and IgG, suggesting that the same B cells may generate a clone that undergoes progressive selection, specialization and class-switching (Grimsholm et al., 2020). In convalescent patients, it has been demonstrated that clones of IgM-, IgG-, and IgA-producing B cells derive from common progenitor cells. In addition, dimeric IgA was more potent than IgA monomers and IgG against the same target (Wang et al., 2020).

Being a mucosal targeted virus, SARS-CoV-2 secretory IgA plays an important role in the early defense and viral containment. As we specified above, IgA serum concentrations decreased one month after the onset of symptoms, while neutralizing IgA remained detectable in saliva for a longer time (Burgess et al., 2020). A human monoclonal IgA antibody cross-reactive with SARS-CoV and SARS-CoV-2 spike proteins was able to neutralize SARS-Cov-2 infection *in vitro* when converted to sIgA (Ejemel et al., 2020). Secretory IgA in the gut and monomeric IgA in the serum are clonally related (Iversen et al., 2017). sIgA specific for SARS-CoV-2 has been detected in the milk (Fox et al., 2020) of SARS-CoV-2 infected mothers, thus demonstrating that sIgA is produced in response to the infection and it is passed to the neonate for protection. It has been suggested that also in adults the measurement of SARS-CoV-2 sIgA might help to identify those individuals that, protected by sIgA, did not develop symptoms and disease (Burgess et al., 2020).

## Primary Antibody Deficiencies, Selective IgA Deficiency and COVID 19

The impact of the COVID-19 pandemic on patients with rare diseases is still not well defined. Inborn Errors of Immunity (IEI) encompasses a group of rare disorders characterized by congenital defects of one or more components of the immune system (Picard et al., 2018), and as such they may be considered as groups at high risk during this pandemic. Selective deficiencies of specific components of the immune response in humans might help dissect and analyze the physiologic role and the outcomes in the interactions with pathogens. The lack of antibodies associated with protection might represent a possible cause of COVID-19 severity, and vaccine failure in particular in patients with primary antibody deficiencies (PAD). We have already reported (Meyts et al., 2020; Quinti et al., 2020; Quinti et al., 2021) on patients affected by PAD lacking antibodies showing a different COVID-19 clinical course. In the most common form of symptomatic PAD - the Common Variable Immune Deficiencies, COVID-19 might have, as expected, a severe clinical course, while in patients with X-linked Agammaglobulinemia (XLA) SARS-Cov-2 infection might remain asymptomatic or have a mild disease. Both CVID and XLA have a severe hypogammaglobulinemia and a defect in antibody production. However, XLA patients only lack B cells due a BTK mutation with consequent arrest of B cell development in the bone marrow. We then speculated on a possible harmful role of B cells in COVID-19 pathogenesis and our hypothesis was further supported by the demonstration that patients under BTK inhibitors also had a mild COVID-19. However, our hypothesis was argued since other agammaglobulinemic patients later reported in the literature showed an aggressive COVID-19 disease, even if none died. This extreme variability in the context of a defective immune response may depend on various factors that need to be defined on larger cohorts of affected patients, since a low number of IEI infected patients with variable outcome has been reported until now. This low number of infected patients might be explained by the choice of most physicians involved in the management of IEI patients to inform early in the pandemic about safety measures, to switch most of them to home therapy and to remote assistance (Pulvirenti et al., 2020b). However, it should be mentioned here that each PAD patient represents a unique model in that the COVID-19 clinical expression varies also within the same immunodeficiency entity, as we knew, from many other comorbidities, patients with primary immune deficiency faced during their life.

Selective IgA Deficiency (SIgAD) is a primary immunodeficiency characterized by an undetectable level of immunoglobulin A in the blood and secretions but no other immunoglobulin deficiencies (Picard et al., 2018). The role defined for IgA antibodies in SARS-Cov-2 protection, offers the possibility to address important aspects of the interplay between SARS-CoV-2 infection and prevention in patients with SIgAD.

It has been shown that a low frequency of SIgAD has been shown to positively correlate with the lower prevalence of COVID-19 in Japan in comparison to other countries where SIgAD reaches a frequency of 1:600 inhabitants (Naito et al., 2020). However, differently from what suggested in the paper, there is not the strategy to promote the production of serum and sIgA in patients with Selective IgA deficiency, nor it is possible to use other therapeutic strategies, such as the use of convalescent plasma, in that, in patients who lack IgA there is a contraindication for the use of plasma for possible adverse reactions. Only when IgA are detectable in serum, convalescent plasma might be administered, as suggested in other forms of PAD (Ho et al., 2021).

Moreover, patients with SIgAD are prone to develop autoantibodies (Hammarström et al., 2000; Quinti et al., 2011; Tangye et al., 2020; Pulvirenti et al., 2020a). In an international study on Coronavirus disease 2019 in patients with inborn errors of immunity, one patient with Selective IgA Deficiency and IgG2 subclass deficiency and COVID died for AIHA (Meyts et al., 2020). An additional case study showed a patient affected from SIgAD since childhood who - similarly to the vast majority of Selective IgA Deficiency - was asymptomatic until a Guillain Barrè syndrome was diagnosed at the time of COVID-19, when she developed anti-ganglioside antibodies (Pfeuffer et al., 2020). The pathogenic role of autoantibodies in severe COVID-19 was further demonstrated by a study showing that clinically silent anti-IFN type I neutralizing antibodies were present in about 10% of patients who develop a severe COVID-19 and in less than 0.1% of asymptomatic patients and of healthy individuals (Bastard et al., 2020).

Lastly, PAD patients and SIgAD patients might have a very low or suboptimal response to SARS-CoV-2 immunization, lacking a major tool for systemic and mucosal protection. It has been reported that patients of PAD (Naito et al., 2020), have an increased length of SARS-CoV-2 positivity, before their swab becomes PCR negative, even if this finding may be observed even in the general population of non-immunocompromised subjects. This finding is in fact in agreement with the observation that immunocompromised patients and severe-to-critical patients may remain a source of infection to their environment (Choi et al., 2020). This prolonged viral shedding represents a potential risk for the spreading of the virus in the community, and for virus genetic changes since high rates of mutation might arise, as previously suggested in studies on patients with Primary Immune Deficiencies chronically infected with other viruses, such as chronic OPV infections (Aghamohammadi et al., 2017).

In conclusion, the strikingly different clinical course of COVID-19 in patients affected with different antibody deficiencies, including the SIgAD entity, requires in-depth studies. PAD are rare diseases while Selective IgA Deficiency is the most frequent antibody deficiency, often undiagnosed for the paucity of symptoms. We suggest to promote studies to clarify the open questions raised on possible consequences of the lack of an IgA response to SARS-CoV-2, since patients affected by primary defects of immunity might always represent a fundamental group to understand the pathogenic mechanisms underlying most infectious and inflammatory diseases.

## Author Contributions

IQ and RC planned and wrote the manuscript. EP and CM reviewed the published papers on the topic. All authors contributed to the discussion of data. All authors contributed to the article and approved the submitted version.

## Funding

This work was founded by Sapienza, Progetto Ateneo 2020.

## Conflict of Interest

The authors declare that the research was conducted in the absence of any commercial or financial relationships that could be construed as a potential conflict of interest.
